# Pharmacologic or genetic targeting of peripheral nerves prevents peri-articular traumatic heterotopic ossification

**DOI:** 10.1038/s41413-024-00358-0

**Published:** 2024-09-26

**Authors:** Manyu Zhu, Ji-Hye Yea, Zhao Li, Qizhi Qin, Mingxin Xu, Xin Xing, Stefano Negri, Mary Archer, Monisha Mittal, Benjamin Levi, Aaron W. James

**Affiliations:** 1 Department of Pathology, Johns Hopkins University, Baltimore, MD 21205 USA; 2 Orthopedic Unit, University of Verona, Verona, Italy; 3 Department of Surgery, Center for Organogenesis and Trauma, University of Texas Southwestern Medical Center, Dallas, TX 75390 USA

**Keywords:** Bone, Pathogenesis

## Abstract

Heterotopic ossification (HO) is a pathological process that commonly arises following severe polytrauma, characterized by the anomalous differentiation of mesenchymal progenitor cells and resulting in the formation of ectopic bone in non-skeletal tissues. This abnormal bone growth contributes to pain and reduced mobility, especially when adjacent to a joint. Our prior observations suggested an essential role of NGF (Nerve Growth Factor)-responsive TrkA (Tropomyosin Receptor Kinase A)-expressing peripheral nerves in regulating abnormal osteochondral differentiation following tendon injury. Here, we utilized a recently developed mouse model of hip arthroplasty-induced HO to further validate the role of peripheral nerve regulation of traumatic HO. Nerve ingrowth was either modulated using a knockin transgenic animals with point mutation in TrkA, or local treatment with an FDA-approved formulation of long acting Bupivacaine which prevents peripheral nerve growth. Results demonstrate exuberant sensory and sympathetic nerve growth within the peri-articular HO site, and that both methods to reduce local innervation significantly reduced heterotopic bone formation. TrkA inhibition led to a 34% reduction in bone volume, while bupivacaine treatment resulted in a 50% decrease. Mechanistically, alterations in TGFβ and FGF signaling activation accompanied both methods of local denervation, and a shift in macrophages from M1 to M2 phenotypes was observed. In sum, these studies reinforce the observations that peripheral nerves play a role in the etiopathogenesis of HO, and that targeting local nerves represents a potential therapeutic approach for disease prevention.

## Introduction

Heterotopic ossification (HO) is a process characterized by the formation of bone in non-skeletal tissues, such as muscles and soft tissues. This disorder can be caused by various factors, including surgery, neurological injuries, arthropathies, and genetic disorders.^1^ HO is particularly common around the hip joint, with studies reporting an incidence as high as 50% in patients with femoral fractures and over 65% in those with war-related explosive and high energy injuries.^2^ Moreover, neurogenic HO (NHO) impacts roughly one in five individuals with spinal cord or traumatic brain injury. It predominantly arises around the joints, particularly the hip and elbow.^3^ The occurrence of HO after Total Hip Arthroplasty (THA) varies widely, with approximately 43% of patients showing some degree of HO. However, in high-risk populations, the incidence can exceed 90%. While most cases of HO are mild to moderate in extent, around 9% of cases are severe.^4^ HO can restrict movement and progress to ankylosis, often necessitating surgical intervention.^5^ Despite the frequent occurrence of HO and its associated complications, prophylaxis against HO has traditionally involved low dose radiation therapy or the use of nonsteroidal anti-inflammatory drugs (NSAIDs). Some new therapeutic strategies targeting the pathological processes of HO are currently being investigated, but there are no proven effective novel biological or pharmacological approaches for preventing HO in clinical practice.^6,7^ The limited understanding of the cellular and molecular mechanisms underlying HO formation poses a significant barrier to the identification of new therapeutics.

The influence of neural signaling on ectopic bone formation is increasingly recognized in both clinical observations and animal experiments. Clinical observations have shown that nerve dysfunction can delay skeletal repair,^8^ indicating a direct regulatory role of nerve signaling in bone repair. Animal experiments have also supported these findings. For example, rats with sciatic nerve resection exhibit defective callus formation,^9^ and mice with inferior alveolar denervation show impaired regeneration of mandibular bone defects.^10^ Previous studies from our group have demonstrated that disruption of TrkA signaling, a receptor involved in nerve growth factor (NGF) signaling, hinders angiogenesis and delays callus formation in mice.^11^ These findings highlight the significance of sensory nerves as key regulators of bone formation and regeneration following injury. In a mouse model of HO induced by intramuscular cell injection, Salisbury *et al*. demonstrated that activated sensory nerves contribute to HO development, and inhibiting nerve activation significantly reduces HO formation.^12^ In a recent study conducted by our group, we observed that axonal ingrowth at sites of soft-tissue trauma is an early inciting factor in trauma-induced heterotopic bone formation. Surgical denervation impeded axonal ingrowth, resulting in significant delays in cartilage and bone formation at the HO site.^13^ Furthermore, the suppression of NGF-TrkA signaling, either pharmacologically or genetically, attenuated osteocartilaginous differentiation and the progression of HO.^13^ Liposomal bupivacaine presents a novel modality for local anesthetic administration, circumventing the requirement for catheter placement. Research has indicated its efficacy in THA, resulting in diminished postoperative opioid consumption and shortened patients’ hospital stays. However, its effect on reducing HO remains to be explored.^14^

The present study further underscores the critical role of TrkA-expressing peripheral nerves in the etiopathogenesis of HO. Utilizing the arthroplasty inducing HO model, we observed that targeting peripheral nerves either genetically or chemically led to dramatic reductions in HO formation. Mechanistically, alterations in TGFβ and FGF signaling activation accompanied both methods of local denervation, and a shift in macrophages from M1 to M2 phenotypes was observed. Overall, our findings suggest potential therapeutic avenues for treating HO using FDA approved medications to target peripheral sensory neurons.

## Results

### Acetabular reaming induces peri-articular heterotopic ossification of the hip joint

**Fig. 1:**
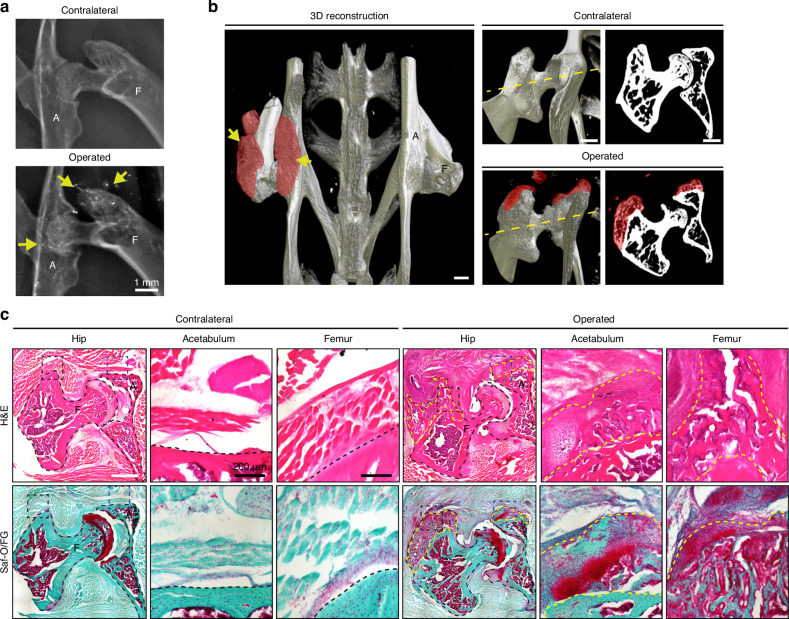
Characterization of hip heterotopic ossification after acetabular reaming. 8 weeks old male and female mice were subjected to acetabular reaming to induce HO, samples were collected at 3 weeks post-operative. **a** Representative roentgenography (XR) image of contralateral and operated hip at 3 weeks post-operative. Yellow arrows indicate HO. A Acetabulum, F Femur. **b** Representative image of reconstructed pelvic microcomputed tomography (µCT) scan shown from a posterior perspective. **c** H&E and Safranin O/Fast green staining. Dashed black lines indicate the margins of the acetabulum and femur and dashed yellow lines indicate the HO site. Cartilage appears orange

Acetabular reaming was employed to induce HO in mouse hip arthroplasty as previously described.^15^ After 3 weeks, XR images revealed notable postoperative HO formation (Fig. 1a). After surgery, mice displayed a mean modified Brooker score of 1.46, indicating moderate heterotopic bone formation (Fig. S1a). Three-dimensional micro computed tomography (μCT) reconstructions of a representative pelvic bone revealed abundant peri-articular heterotopic bone in the acetabulum and femur regions of the operated hip (Fig. 1b). The extent and variability of the disease burden were further evaluated through quantitative μCT analysis. In contrast to the contralateral hip, the operated hip demonstrated significant increase across bone surface (10^8^ μm^2^ increase) and bone volume (2.5 × 10^9^ μm^3^ increase) (Fig. S1b, further separated by anatomical region in Fig. S1c–e). H&E and Safranin O/Fast green staining revealed structural alterations and abnormal bone and cartilage formation in the acetabular and femoral regions (Fig. 1c). Histomorphometric quantification of the operated acetabular and femoral regions showed a significant increase in cartilage area and bone area (Fig. S1f). These results confirm significant endochondral bone formation and HO 3 weeks after acetabular reaming.

### Peripheral nerve growth accompanies peri-articular heterotopic ossification

**Fig. 2:**
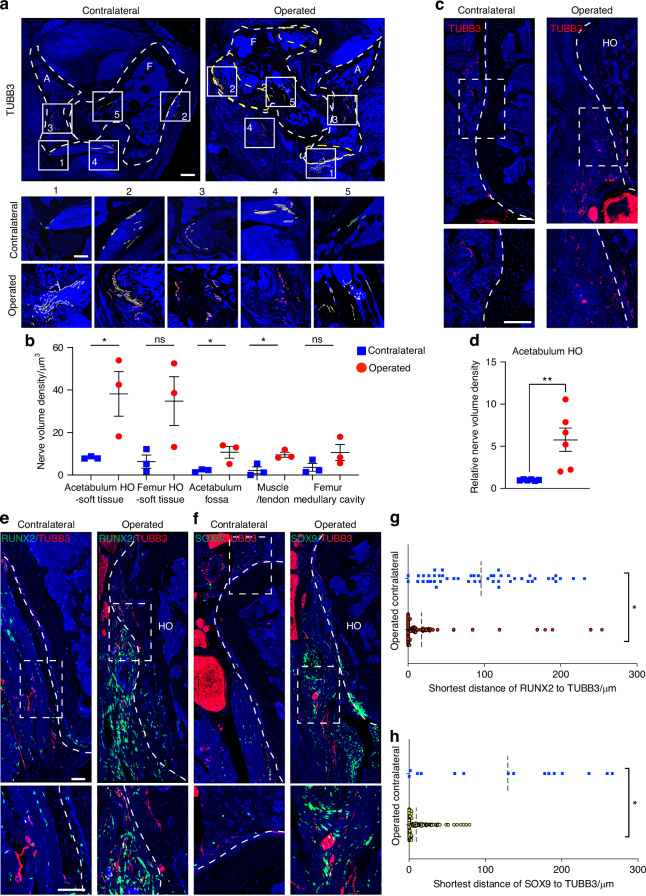
Axonal invasion accompanies hip heterotopic ossification. **a** Innervation interactions between HO and other tissue regions shown by pan-neuronal Beta III Tubulin (TUBB3) immunohistochemical staining. Dashed white lines indicate the margins of the acetabulum and femur. Dashed yellow lines indicate HO site. White boxes indicate five separately analyzed regions of HO. Box 1: acetabulum and surrounding soft tissue. Box 2: femur and surrounding soft tissue. Box 3: acetabulum fossa. Box 4: muscle or tendon. Box 5: femur medullary cavity. **b** Quantification of TUBB3^+^ nerve density changes within five separately analyzed regions, 3 weeks after acetabular reaming (*n* = 3). **c**, **d** Representative image and quantification of TUBB3^+^ nerves within acetabulum associated HO. Tile scans above, while high magnification images are below. **e** Representative images of RUNX2 and TUBB3 co-immunofluorescent staining within the acetabulum associated HO, in comparison to the contralateral side (*n* = 6). **f** Representative images of SOX9 and TUBB3 staining co-immunofluorescent staining within the acetabulum associated HO, in comparison to the contralateral side. **g** Spatial proximity of RUNX2 and TUBB3 immunostaining within the acetabulum associated HO in comparison to the contralateral side. Each dot represents nearest distance of each RUNX2 positive cell to a single TUBB3^+^ nerve fiber. **h** Spatial proximity of SOX9 and TUBB3 in the acetabulum associated HO, in comparison to the contralateral side. Each dot represents nearest distance of each SOX9 positive cell to a single TUBB3^+^ nerve fiber. Scale bars: 500 μm in A the upper images, 1 mm in **a** the magnified images below, 200 μm in **c** and **e**. Data presented as mean ± 1 SD. Dots in scatterplots represent individual measurements. Unpaired two-tailed Student’s *t* test was used for a two-group comparison. ns: not significant, *P < 0.05, **P < 0.01

In our prior studies, we observed that peripheral nerves regulate osteochondral differentiation in a mouse model of HO induced by soft tissue trauma.^13^ Moreover, we found that various soft tissues could undergo ossification following hip arthroplasty.^15^ Here, to investigate the role of peripheral nerves in the formation of heterotopic bone after 3 weeks acetabular reaming, we examined the spatial patterns of innervation interactions between HO and other tissue regions. Immunohistochemical staining for pan-neuronal Beta III tubulin (TUBB3) revealed a significant increase in TUBB3^+^ nerve fibers area (Fig. S2a). Additionally, in an analysis of TUBB3^+^ nerve fiber parameters, it was observed that the width of these nerve fibers stayed constant, while the length showed a significant increase (Fig. S2b, c). To assess the specific location of innervated HO formation after acetabular reaming, we evaluated the TUBB3^+^ nerve fibers density in various post-surgery HO areas, including the acetabulum and peripheral soft tissues, femur and peripheral soft tissues, acetabular fossa, muscle or tendon, and femoral marrow cavity. The results suggested an elevated density of TUBB3^+^ nerve fibers in all areas (Fig. 2a). However, the significant increase was observed mostly in acetabulum related areas, such as acetabulum and surrounding soft tissues (3.69-fold increase), acetabulum fossa (4.13-fold increase), and muscle or tendon (3.36-fold increase) (Fig. 2b). Therefore, to further investigate the neuro-modulatory mechanisms, we focused on innervation changes of acetabulum associated HO in subsequent portions of the study. Analysis in a separate cohort of animals revealed a notable 4.75-fold increase in TUBB3^+^ nerve density was observed to invade the operated acetabular HO compared to the contralateral side (Fig. 2c, d). Furthermore, the distribution of Scx-GFP^+^ stromal cells exhibited an increase in the proximity of the acetabular HO, consistent with our previous observations that Scx expressing cells mark the areas that give rise to heterotopic bone^15^ (Fig. S2d). Next, to examine nerve and osteochondral interactions, co-staining was conducted for the osteoblast marker Runt-related transcription factor 2 (RUNX2) and TUBB3 (Fig. 2e). Results showed significant proximity between RUNX2^+^ osteoblastic cells and TUBB3^+^ nerve fibers. Similar experiments were performed using the chondrocyte marker SRY-Box Transcription Factor 9 (SOX9) and TUBB3 co-staining (Fig. 2f). Similarly, results showed significant spatial proximity between SOX9^+^ chondroblastic cells and TUBB3^+^ nerve fibers. Quantification of distance between RUNX2^+^ or SOX9^+^ cells and the nearest TUBB3^+^ nerve fiber confirmed the overall close spatial association of osteochondral cells and nerves within the HO site in comparison to the uninjured contralateral side (Fig. 2g, h). Thus, peripheral nerve ingrowth is a consistent feature of HO of the hip after an arthroplasty-like procedure and occurs in close spatial proximity to cells of osteochondral differentiation.

### Inhibition of TrkA catalytic activity inhibits peri-articular HO formation

**Fig. 3:**
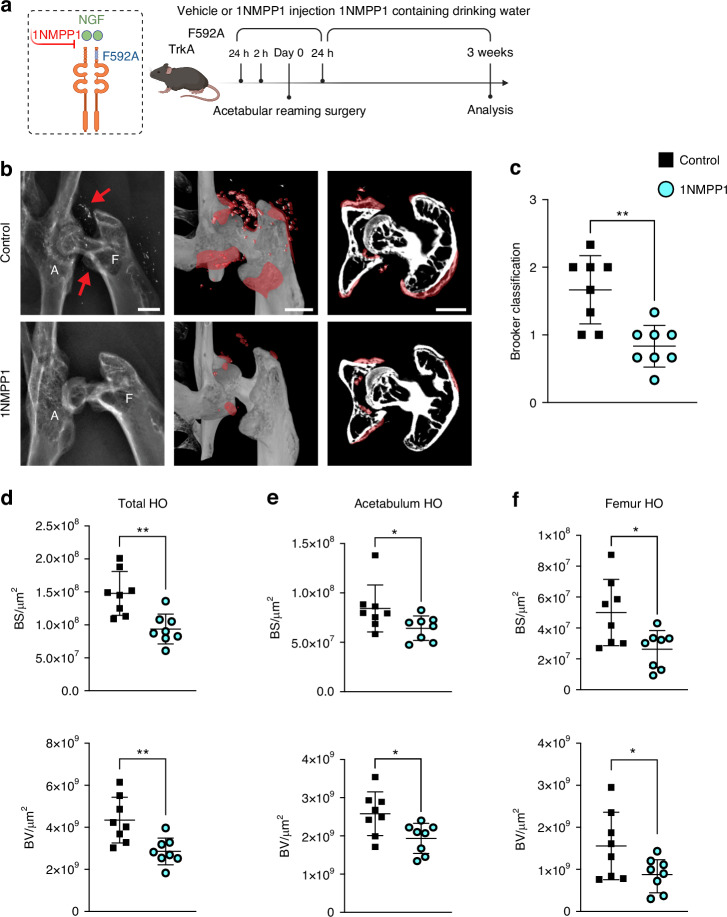
TrkA inhibition in transgenic TrkA^F592A^ mice reduces hip associated HO by radiology. **a** Schematic of the experiment: TrkA^F592A^ mice, susceptible to controllable TrkA inhibition via the small molecule 1NMPP1, were used. TrkA^F592A^ animals underwent HO induction, receiving either 1NMPP1 or vehicle control by i.p. injection 24 h and 2 h pre-operation and 1NMPP1 by drinking water throughout the study. Mice were analyzed 3 weeks post-operative. **b** Representative roentgenography (XR) and microcomputed tomography (µCT) images of control and 1NMPP1 treated TrkA^F592A^ mice. Red arrows and red colorization indicate HO areas. **c** Grading of HO after 1NMPP1 treatment according to a modified Brooker classification. **d** Quantitative μCT analysis of the total HO, including bone surface (BS), bone volume (BV). **e** Quantitative μCT analysis of the acetabulum associated HO. **f** Quantitative μCT analysis of femur associated HO. Dots in scatterplots represent an individual animal. *n* = 8. Scale bars: 1 mm. Data presented as mean ± 1 SD. Unpaired two-tailed Student’s *t* test was used for a two-group comparison. *P < 0.05, **P < 0.01

Having identified prominent changes in local innervation associated with peri-articular HO development, we next set to determine the extent to which methods to inhibit nerve growth may influence disease burden. First, a previously validated knockin transgenic mouse that also carries a *Thy1*-YFP reporter was utilized, in which TrkA signaling is acutely disrupted in TrkA^F592A^ by administration of the small molecule 1NMPP1, and *Thy1*-YFP is robustly expressed in all peripheral nerves^13,16^ (Fig. 3a). This approach has been validated by our group in several orthopaedic models, and the small molecule 1NMPP1 has no known skeletal or neural effects unless animals have the TrkA^F592A^ allele. As per prior studies, TrkA^F592A^ mice were pretreated with 1NMPP1 or DMSO as control, and then subjected to acetabular reaming (Fig. 3a). HO formation was confirmed by X-ray and µCT reconstruction (Fig. 3b) among control and 1NMPP1 treated TrkA^F592A^ mice. The modified Brooker classification revealed a significant reduction in HO formation among 1NMPP1 treated mice (Fig. 3c). HO burden was further assessed by quantitative µCT analysis (Fig. 3d-f). Assessments were performed separately in total HO (Fig. 3d), acetabulum associated HO (Fig. 3e), and femur associated HO (Fig. 3f) and analyses included bone surface (BS) and bone volume (BV). In total HO, the operated hip showed significant reduction in BS (36.6% reduction), BV (34.3% reduction) (Fig. 3d). Similarly in acetabulum and femur HO, a significant decrease in BS and BV were observed in 1NMPP1 treated mice (Fig. 3e, f). Together, our data suggests that inhibition of TrkA^+^ nerves via a chemical-genetic approach significantly blunts HO formation.

**Fig. 4:**
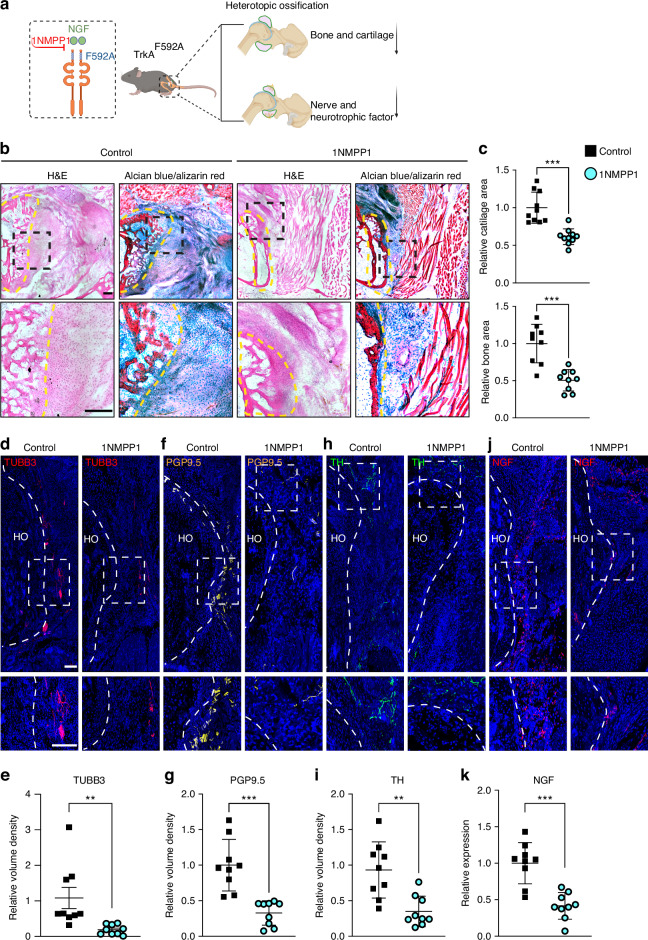
TrkA inhibition in transgenic TrkA^F592A^ mice reduces hip associated HO by histology. **a** Schematic of the experiment. Mice were treated with 1NMPP1 or vehicle control throughout the study period. HO and nerve analysis at 3 weeks post-operative. **b** H&E and Alcian Blue/Alizarin Red staining of HO. Dashed black lines indicate the high magnification area of the acetabulum and dashed yellow lines indicate HO site. Cartilage appears blue and bone appears red. **c** Quantification of bone and cartilaginous area within the HO site. *n* = 9 animals per group. **d**, **e** Representative images and quantification of immunostaining of TUBB3^+^ nerves within acetabulum associated HO. Tile scans appear above, while high magnification images are below. **f**, **g** Representative images and quantification of immunostains for the sensory nerve marker PGP9.5 within acetabulum associated HO. **h**, **i** Representative images and quantification of immunostains for the sympathetic nerve marker tyrosine hydroxylase (TH). **j**, **k** Representative images and quantification of immunostaining for the neurotrophin nerve growth factor (NGF). Scale bars: 200 μm. Data presented as mean ± 1 SD. No differences were observed any parameter examined between male and female animals. Dots in scatterplots represent an individual animal. Unpaired two-tailed Student’s *t* test was used for a two-group comparison. **P < 0.01, ***P < 0.001

To explore the impact of TrkA inhibition on HO site innervation and osteochondral formation following acetabular reaming, we used TrkA^F592A^/*Thy1*-YFP reporter mice and analyzed bone and cartilage formation through histologic stains. Additionally, we assessed the expression of nerve markers and nerve growth factor (NGF), the primary neurotrophin that stimulates sensory nerve ingrowth,^17^ using immunostaining (Fig. 4a). Consistent with µCT results, H&E and Alcian Blue/Alizarin Red staining revealed a notable decrease in bone and cartilage formation in the peri-acetabular region in 1NMPP1-treated TrkA^F592A^ mice in comparison to control (Fig. 4b, c, 38.5% decrease in cartilage and 49.5% decrease in bone area). Next, the changes in innervation within the acetabular HO site were examined using the pan-neuronal marker TUBB3. At 3 weeks post-injury, TUBB3^+^ nerve fiber staining was significantly reduced by 89.2% after treatment with 1NMPP1 (Fig. 4d, e), with substantial overlap with *Thy1* reporter activity (Fig. S3).^18^ Characterization of nerve fiber type was subsequently conducted through immunohistochemical detection, using either the sensory nerve marker PGP9.5 or the sympathetic marker tyrosine hydroxylase (TH). Visually, PGP9.5^+^ appeared significantly more abundant than TH^+^ fibers within the control-treated HO site (Fig. 4f, h). Results showed a substantial reduction in both PGP9.5^+^ and TH^+^ nerves was evident in the 1NMPP1 treatment group (Fig. 4f, h), displaying similar reductions of 67.3% and 62.5%, respectively (Fig. 4g, i). Interestingly, NGF immunostaining showed similar changes within 1NMPP1 treated mice, revealing a 58.4% decrease in among TrkA inhibited mice (Fig. 4j, k). In aggregate, these results demonstrate that chemical-genetic inhibition of the NGF-TrkA signaling axis suppresses sensory and sympathetic nerve growth as well as osteochondral differentiation in peri-articular HO.

### An FDA-approved formulation of bupivacaine inhibits peri-articular HO formation

**Fig. 5:**
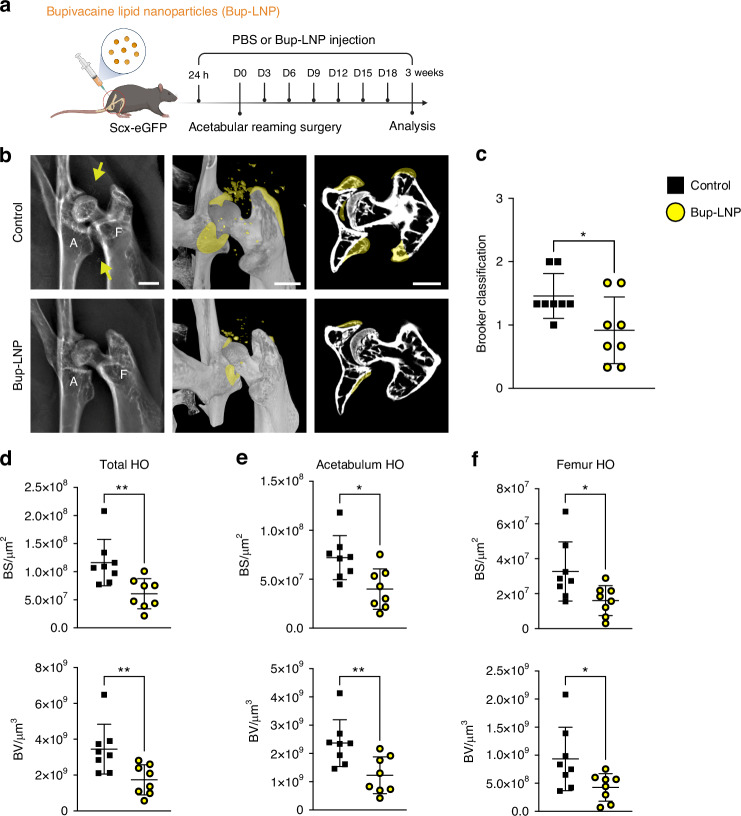
Bupivacaine lipid nanoparticles inhibit hip associated HO by radiology. **a** Schematic diagram of the experiment: Scx-eGFP mice received local injections of bupivacaine lipid nanoparticles (Bup-LNP) and PBS control 24 h before surgery and every 3 d after acetabular reaming. Mice were analyzed 3 weeks post-operative. **b** Representative roentgenography (XR) and microcomputed tomography (µCT) images of Bup-LNP and control treated animals. Yellow arrows and yellow colorization highlight HO areas. **c** Grading of HO according to a modified Brooker classification. **d** Quantitative μCT analysis of total HO, including bone surface (BS) and bone volume (BV). **e** Quantitative μCT analysis of the acetabulum associated HO. **f** Quantitative μCT analysis of femur associated HO. Scale bars: 1 mm. Data presented as mean ± 1 SD. Dots in scatterplots represent an individual animal. Unpaired two-tailed Student’s *t* test was used for a two-group comparison. *P < 0.05, **P < 0.01

To explore the translational potential of targeting peripheral nerves in HO, we utilized a bupivacaine lipid nanoparticle system (Bup-LNP), an FDA approved long-acting local analgesic. Bupivacaine is well established to inhibit peripheral axon growth, along with its analgesic effects.^19^ Scx-eGFP reporter mice were used, and Bup-LNPs were applied 24 h before surgery and every 3 days afterward, according to the established clinical half-life^20^ (Fig. 5a). HO formation was next assessed by X-ray and µCT reconstruction among Bup-LNP and PBS treated mice (Fig. 5b). Modified Brooker classification showed a significant reduction in HO formation among Bup-LNP treated mice (Fig. 5c). HO burden was further assessed by quantitative µCT analysis (Fig. 5d–f). Assessments were performed separately in total HO (Fig. 5d), acetabulum associated HO (Fig. 5e), and femur associated HO (Fig. 5f). In total HO, the treated hip led to a significant improvement in nearly all quantitative metrics in comparison to control mice, including BS (47.7% reduction) and BV (49.6% reduction) (Fig. 5d). Similar effects were observed in acetabulum-associated HO, where Bup-LNP treatment resulted in significant reduction in BS (44.5% reduction) and BV (48.1% reduction) (Fig. 5e). Among femur-associated HO, decreased BS and BV were also observed (Fig. 5f). Together, these findings suggest that Bup-LNP potently prevents peri-articular HO formation.

**Fig. 6:**
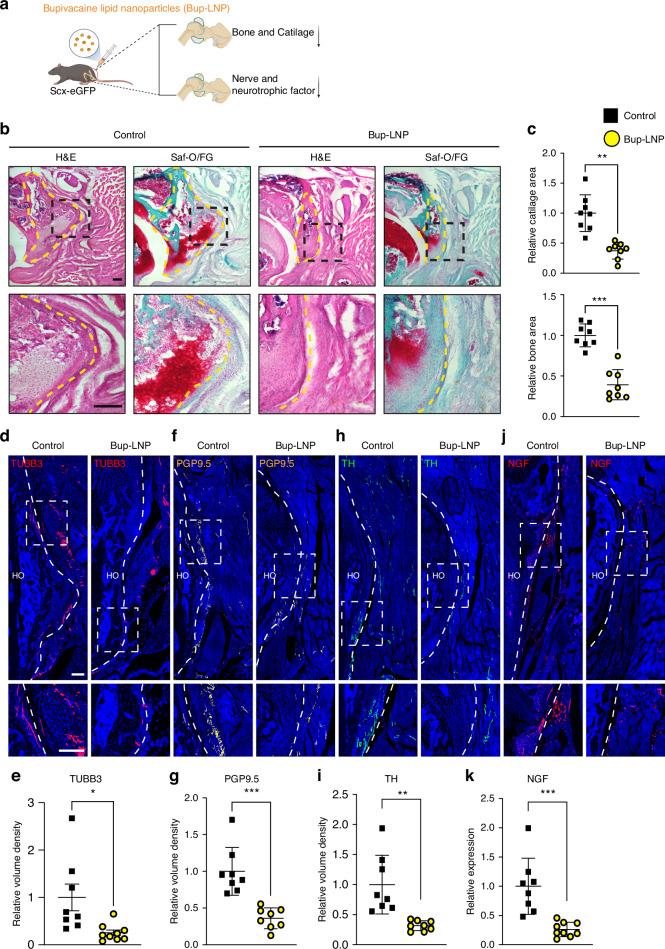
Bupivacaine lipid nanoparticles inhibit hip associated HO by histology. **a** Schematic diagram of the experiment: Scx-eGFP mice received local injections of bupivacaine lipid nanoparticles (Bup-LNP) and PBS control 24 h before surgery and every 3 d after acetabular reaming. Mice were analyzed 3 weeks post-operative. **b** H&E and Safranin O/Fast green staining of HO. Dashed black lines indicate the high magnification area of the acetabulum and dashed yellow lines indicate HO site. Cartilage appears orange. **c** Quantification of bone and cartilaginous area within the HO site. *n* = 8 animals per group. **d**, **e** Representative images and quantification of TUBB3 immunostaining within acetabulum associated HO. Tile scans appear above, while high magnification images are below. **f**, **g** Representative images and quantification of PGP9.5 immunostaining. **h**, **i** Representative images and quantification of tyrosine hydroxylase (TH) immunostaining. **j**, **k** Representative images and quantification of the neurotrophin nerve growth factor (NGF). Scale bars: 200 μm. Data presented as mean ± 1 SD. No differences were observed any parameter examined between male and female animals. Dots in scatterplots represent an individual measurement. Unpaired two-tailed Student’s *t* test was used for a two-group comparison. *P < 0.05, **P < 0.01, ***P < 0.001

We next assessed bone and cartilage formation through histologic stains and examined the patterns of innervation and NGF using immunostaining (Fig. 6a). In similarity to treated TrkA^F592A^ mice, H&E and Safranin-O/Fast green staining revealed a notable decrease in aberrant bone and cartilage formation in Bup-LNP treated animals (Fig. 6b, c, 62.5% and 60.8% reduction in cartilage and bone area, respectively). Furthermore, the changes in nerve fibers within the acetabular HO site were initially examined using the pan-neuronal marker TUBB3. A significant decrease in TUBB3^+^ nerve fibers was observed within the HO site following Bup-LNP treatment (Fig. 6d, e, 74.7% reduction). In addition, the distribution of Scx-GFP^+^ stromal cells was similarly reduced in the vicinity of the acetabular HO (Fig. S6). The characterization of nerve fiber type was subsequently conducted through immunohistochemical detection, again using either PGP9.5 or TH. Results showed a substantial reduction in both PGP9.5^+^ and TH^+^ nerves within Bup-LNP treated samples (Fig. 6f, h), displaying reductions of 63.9% and 68.3%, respectively (Fig. 6g, i). NGF staining was likewise reduced, showing a 73.9% decrease in Bup-LNP treated HO sites (Fig. 6j, k). In combination, the interruption of nerve impulse transmission and nerve growth via Bup-LNP treatment effectively suppressed both local sensory and sympathetic nerve sprouting and ultimately potently suppressed peri-articular HO.

### Reduced innervation is associated with a shift from TGFβ to FGF signaling activation

**Fig. 7:**
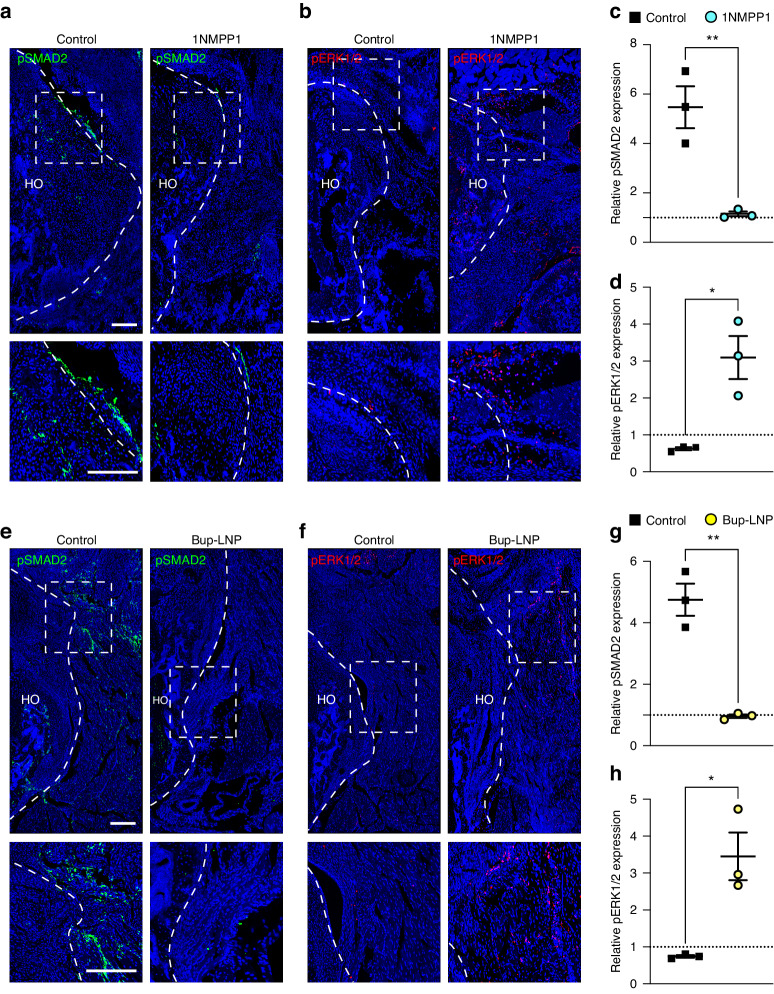
Reduced HO site innervation is associated with altered TGFβ and FGF signaling activation. **a** Representative images of pSMAD2 immunofluorescent staining within acetabulum associated HO in TrkA^F592A^ mice treated with either 1NMPP1 or vehicle control. Tile scans appear above, while high magnification images are below. **b** Representative images of pERK1/2 immunofluorescent staining in TrkA^F592A^ mice treated with either 1NMPP1 or vehicle control. **c**, **d** Quantification of pSMAD2 and pERK1/2 expression immunostaining among HO in TrkA^F592A^ mice treated with either 1NMPP1 or vehicle control. Each dot represents an individual animal, and each group is normalized to the non-operated control. **e** Representative images of pSMAD2 immunofluorescent staining within acetabulum associated HO among mice treated with Bup-LNP or PBS control. Tile scans appear above, while high magnification images are below. **f** Representative images of pERK1/2 immunofluorescent staining within mice treated with Bup-LNP or PBS control. **g**, **h** Quantification of pSMAD2 and pERK1/2 expression after Bup-LNP treatment versus PBS control. Each dot represents an individual animal, and each group is normalized to the non-operated control. *n* = 3 per group. Scale bars: 200 μm. Data presented as mean ± 1 SD. Unpaired two-tailed Student’s *t* test was used for a two-group comparison. *P < 0.05, **P < 0.01

While both interventions used here to prevent peripheral nerve growth successfully reduced peri-articular HO formation, the underlying molecular coupling mechanisms of neurogenesis and osteochondrogenesis in HO remain unclear. In our prior study using the injured Achilles tendon as a model, surgical denervation led to a transition from TGFβ to FGF signaling activation in pre-chondrogenic cells.^13^ Building on these observations, immunohistochemical staining for downstream markers of TGFβ and FGF signaling activation were assessed in peri-acetabular tissues (pSMAD2 and pERK1/2, respectively). In control treated TrkA^F592A^ mice with intact innervation, immunoreactivity of pSMAD2 was increased in and around the peri-acetabular HO site, present in nuclear and perinuclear locations. (Fig. 7a). However, in 1NMPP1-treated TrkA^F592A^ samples with reduced innervation, a significant diminution in pSMAD2 immunoreactivity was observed (Fig. 7a–c, 78.8% reduction). Contrasting results were obtained with pERK1/2 immunostaining (Fig. 7b–d). Here, pERK1/2 staining revealed a 3.79-fold increase at injury sites within the 1NMPP1-treated group in comparison to vehicle (Fig. 7b–d). In addition, at a later time point, the 1NMPP1-treated group effectively diminished osteochondral markers while also exhibiting the same shift from TGFβ to FGF signaling activation (Fig. S5). These trends in alterations of TGFβ and FGF signaling were remarkably conserved among mice subjected to Bup-LNP treatment as well (Fig. 7e–h). After 3 weeks of surgery, pSMAD2 expression decreased by 79.7% in the Bup-LNP treatment group compared with the PBS control group, whereas pERK1/2 expression increased by 3.64-fold (Fig. 7e–h). Likewise, TGFβ1 immunostaining significantly decreased, whereas FGF2 immunostaining was significantly increased in the Bup-LNP-treated group (Fig. S6). Hence, excessive innervation at the injury site is linked to a shift in local FGF and TGFβ signaling, and our interventions to reduce HO associated nerve ingrowth appear to reverse this pattern of signaling activation.

### Reduced innervation is associated with skewed macrophage polarization in the HO area

**Fig. 8:**
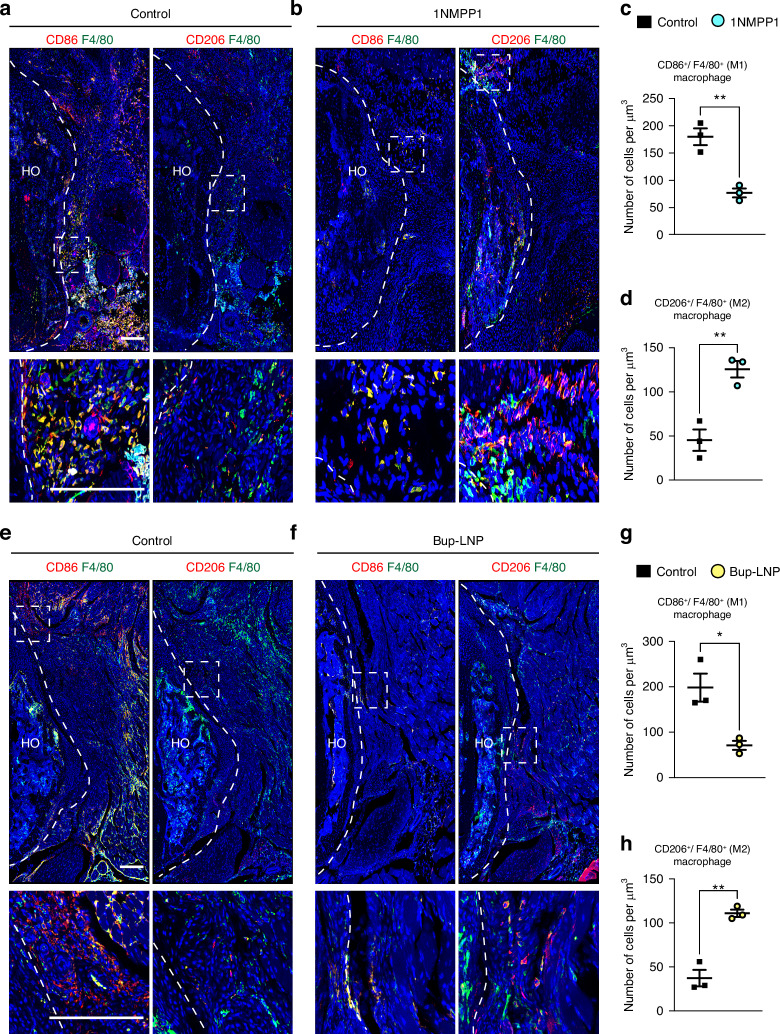
Reduced HO site innervation is associated with polarization of HO infiltrating macrophages. Assessments of macrophage polarization were performed in the acetabulum associated HO areas, 3 weeks post-injury. **a**, **b** Representative images of CD86, CD206 and F4/80 immunofluorescent staining among control or 1NMPP1 treated TrkAF592A mice. Tile scans appear above, while high magnification images are below. **c**, **d** Quantification of CD86^+^/F4/80^+^ (M1-like macrophage) and CD206^+^/F4/80^+^ (M2-like macrophage) cell density among control or 1NMPP1 treated TrkAF592A mice. **e**, **f** Representative images of CD86, CD206 and F4/80 immunofluorescent staining among Bup-LNP or control treated mice. Tile scans appear above, while high magnification images are below. **g**, **h** Quantification of CD86^+^/F4/80^+^ (M1-like macrophage) and CD206^+^/F4/80^+^ (M2-like macrophage) cell density among Bup-LNP or control treated mice. *n* = 3 per group. Scale bars: 200 μm. Data presented as mean ± 1 SD. Dots in scatterplots represent an individual mouse measurement. Unpaired two-tailed Student’s *t* test was used for a two-group comparison. *P < 0.05, **P < 0.01

Inflammation is crucial in HO development, and macrophages play a well-established role in inflammatory regulation of HO genesis.^21,22^ Our prior single-cell RNA sequencing in the context of experimental denervation of HO revealed subtle differences in macrophage phenotypes in tendon associated HO, characterized by a reduction in the expression of M1-like gene profile and a simultaneous increase in the expression of M2-like genes.^13^ Here, the frequency of total macrophages was first assessed using the pan-marker F4/80 (Fig. S7). Across experimental conditions of reduced innervation, the overall density of F4/80^+^ macrophages did not exhibit a significant change (Fig. S7). Next, classic M1 and M2 markers were visualized by immunofluorescent staining (CD86 and CD206, respectively) and co-stained with the pan marker F4/80. In 1NMPP1 treated TrkA^F592A^ mice, the density of CD86^+^ F4/80^+^ M1-like macrophages was 57.2% lower in 1NMPP1-treated animals (Fig. 8a–c). Conversely, immunohistochemical staining of CD206^+^/F4/80^+^ M2-like macrophages demonstrated a 1.77-fold increase at the injury site in the 1NMPP1-treated animals compared with the control group (Fig. 8b–d). This pattern was also observed among the Bup-LNP-treated samples. Here, the number of M1-like macrophages in the Bup-LNP-treated group decreased by 64.2% in comparison to the control group, while the number of M2-like macrophages increased by 1.97-fold (Fig. 8e–h). Thus, inhibition of HO associated peripheral nerves by independent methods did not appear to alter macrophage frequency, but did result in macrophage repolarization from M1-like to M2-like within the injured tissues.

## Discussion

In this study, we propose a novel approach for the translational therapy of HO. We examined the crucial role of nerves in the abnormal cartilage and bone formation induced by acetabular reaming. We found that inhibiting the TrkA signaling pathway demonstrated the capacity to reduce ectopic osteochondral formation after injury. Additionally, we noted that mice treated with Bup-LNP effectively reduced the formation of hip HO after injury. Downstream cellular and molecular alterations were observed with interventions to reduce innervation, including changes in macrophage immunophenotype and TGFβ / FGF signaling activation. These findings have direct relevance for trauma-induced HO, and may in fact also be relevant for NHO, in which neuronal injury initiates neurogenic inflammation, resulting in the release of neuropeptides into the peripheral circulation.^23^

Bupivacaine and related analgesics are well established to have several effects on the peripheral nervous system.^24,25^ Bupivacaine, a non-opioid agent known for its selective blockade of sodium channels, demonstrates efficacy in halting nerve impulse propagation and mitigating pain signaling.^24^ However, its documented adverse effects include neurotoxicity and inhibition of axonal growth and regeneration.^19,26,27^ Bupivacaine is commonly used for local infiltration analgesia following hip replacement surgery,^28^ albeit only within the surgical period and not for HO prophylaxis. FDA-approved long acting liposomal formulations of bupivacaine as used in the current study (Exparel®) have been FDA approved since 2011^29^ and are most commonly used in spinal block procedures.^30^ Kaduri et al. demonstrated the use of such bupivacaine lipid nanoparticles can suppress nerve growth in breast cancer models and as a consequence suppress neural-tumor paracrine interactions.^20^ This data, along with the present study, highlights the potential of long-acting bupivacaine formulations as novel therapeutic agents with both analgesic and disease-modifying properties. Although not observed in our study, it is important to note that depending on the dose bupivacaine may have myotoxic effects,^31^ which (if present) would logically exacerbate rather than prevent HO.

A crucial question that still requires further investigation is the potential downstream molecular mechanisms by which peripheral nerves within injured soft tissue positively regulate the genesis and/or progression of heterotopic bone. Data to date in other skeletal systems suggest that these represent non-contact dependent paracrine interactions, as for example conditioned medium derived from neurons has potent cellular effects on skeletal cells.^32,33^ In other contexts, investigators have begun to query whether peripheral neural paracrine signaling relies on extracellular vesicles (EVs) or non-EV transmission,^34–38^ a concept that has not yet been formally explored in the context of HO. In terms of individual neural-derived signaling factors, many growth and differentiation factors (GDFs) are found in peripheral nerves that would logically regulate HO. TGFβ isoforms themselves are expressed within sensory nerve ganglia,^39^ and could represent one of several sources of TGFβ that work in concert to positively regulate HO formation.^40^ Pro-angiogenic factors such as PDGFRA and VEGFA have been found within sensory ganglia,^41,42^ and their expression induced in the context of experimental HO models.^43^ Recent experimental work in cranial bone have implicated specific nerve-derived paracrine factors, including the TGFβ modulator FSTL1 as well as FGF1, which is well established to have high expression in sensory nerve ganglia.^33^

Mounting research evidence indicates a close association between macrophages and HO, and macrophage recruitment and activation identified as key drivers of HO.^21^ Research by Genet et al. underscored the pivotal role of F4/80^+^ resident tissue macrophages in driving NHO following spinal cord injury. Ablating these macrophages led to a notable decrease in the volume of NHO.^44^ M1-like macrophages typically initiate an immediate inflammatory response following injury, and during the subsequent inflammatory repair phase, infiltrating macrophages exhibit an M2-like phenotype.^45^ Controllable delivery of neuroinflammatory mediators substance P (SP) and calcitonin gene-related peptide (CGRP) in murine Achilles tendons can modulate the expression of M1-like macrophages, potentially amplifying the inflammatory response and activating TGFβ and BMP signaling, thus triggering HO.^46^ Conversely, the depletion of M2 macrophages has been shown to promote the development of HO.^47,48^ Besides, in vitro studies have shown that bupivacaine-encapsulated mesenchymal stromal cells can secrete higher levels of anti-inflammatory prostaglandin E2 (PGE2), effectively inhibiting M1-like macrophages activity and promoting alternative M2-like macrophages phenotypes.^49,50^ The NGF-TrkA signaling pathway itself has been shown to induce the expression of phagocytic M1-like macrophages.^51^ Additionally, TrkA expression in cultured human macrophages facilitates TGF-β secretion and reduces the expression of CD206, a marker associated with M2-like macrophages.^51^ Our results, in combination with the prior literature on macrophage influence on HO, suggests that intriguing possibility that peripheral nerve regulation of HO development may, at least in part, depend on peripheral nerve regulation of macrophage phenotype.

This present work has certain limitations. Firstly, our findings show HO in both soft tissue and on the periosteal surface post-arthroplasty.^15^ Whilst typically forming in soft tissues, HO can also attach to bone surfaces (termed parosteal HO), potentially leading to joint ankylosis.^52^ However, we cannot exclude that there is potential for overlap in the pathophysiology of parosteal HO with other processes of periosteal bone formation, such as periosteal reactive bone and even osteophytes. Secondly, relatively young animals were used (6–8 weeks old), in similarity to past studies.^13,53^ In future studies, utilization of skeletally mature animals would ensure more direct comparability to HO that occurs clinically. Third, our findings from liposomal bupivacaine treatment were related to repeated exposure to drug. It is possible that upon cessation of bupivacaine treatment, heterotopic bone formation may rebound. Finally, we acknowledge that inhibition of skeletal-innervating nerves and/or TrkA^+^ nerves may cause skeletal side effects. For example, clinical trials involving anti-nerve growth factor (anti-NGF) have been halted due to the emergence of abnormal subchondral fractures and rapidly progressive osteoarthropathy.^54^ Similarly TrkA inhibitors, employed clinically to treat specific cancers, have also been associated with an elevated fracture risk.^55^ We therefore cannot rule out that inhibition of bone-related nerves may pose a fracture risk or other skeletal complications in some cases.

In summary, this study expands our understanding of the role of peripheral nerves in heterotopic bone, suggesting a complex interplay between peripheral nerves, mesenchymal progenitor and immune cells. These findings further suggest that commonly used and FDA approved drugs may have utility for prophylaxis against this common and potentially debilitating disease process.

## Materials and methods

### Animals and conditions

All animal procedures were ethically approved by the Institutional Animal Care and Use Committee (IACUC) of Johns Hopkins University (MO19M366). Animals were housed in IACUC-supervised facilities under controlled environmental conditions, including a temperature range of 18–22 °C, relative humidity of 50% (± 20%), and a 12 h light-dark cycle. Mice were provided ad libitum access to standard laboratory chow and water. Mouse strains utilized in the experiments were either generated in-house or obtained from Jackson Laboratory. TrkA^F592A^ mice, homozygous for a phenylalanine-to-alanine point mutation in exon 12 of the mouse *Ntrk1* gene (F592A), were donated by the Ginty laboratory. This mutation in TrkA^F592A^ mice confers sensitivity to inhibition by the membrane-permeable small-molecule 1NMPP1, resulting in 50% or greater inhibition of TrkA catalytic activity.^17,56^ The Scleraxis (Scx)-GFP reporter animal were used based on our prior research indicating the expression of the Scx-GFP reporter in mesenchymal progenitor cells,^13^ which has previously been validated in the hip post-arthroplasty model.^53^ Scx-GFP reporter animals were generously provided by the Fan Laboratories. In this model, the open reading frame of GFP is inserted into the first exon of the Scx gene.^57^ The experimental cohort consisted of male and female mice aged 6–8 weeks. Littermate analysis, conducted in a blinded manner to genotype, was employed. A total of 42 mice were utilized in the study. *n* = 8 C57BL/6J wild-type mice, *n* = 18 TrkA^F592A^ mice divided into two groups receiving either 1NMPP1 treatment or DMSO as control, and *n* = 16 Scx-GFP mice divided into two groups receiving either Bup-LNP treatment or Phosphate-buffered saline (PBS) as control. All mice underwent acetabular reaming surgeries and were analyzed at 3 weeks post-surgery. A subset of animals was examined at 7 weeks post-surgery. After 3 weeks, roentgenography (XR) images were classified using a modified Brooker system adapted for mice.^15,58^

### Surgical procedures

For the hip post-arthroplasty HO model, the left leg was operated on in all cases. Acetabular reaming surgeries were performed as previously described.^15^ Briefly, animals were anesthetized with inhaled isoflurane (3%–5% induction, 2%–3% maintenance) delivered with combined oxygen and nitrous oxide (1:2 ratio). Sustained-release buprenorphine (1.2 mg/kg SC) was also administered subdermally. The animal was placed in a lateral decubitus position. The hair was clipped, and skin was disinfected with povidone-iodine 5% and alcohol 70%. A 2 cm skin incision was made centered on the greater trochanter and directed proximal to the iliac crest and distally over the lateral shaft of the femur. The joint was reached following the intermuscular plane between the tensor fascia lata and gluteus medius muscles. A capsulotomy was then performed. The femoral head was partially dislocated to enable acetabular reaming with a 1.2 mm diameter reamer (Cell-point Scientific, CITY, STATE) and micropower drill (Roboz Surgical Instrument Co.). The surgical site was irrigated with saline solution. The gluteus medius and tensor fascia lata were then re-approximated with Vicryl 4-0 suture (Ethicon Inc, Raritan, NJ) and the skin was then closed with Prolene 5-0 suture (Ethicon Inc. Raritan, NJ). Samples were collected at 3 weeks post-injury for analysis.

To temporally inhibit TrkA catalytic activity in TrkA^F592A^ animals, we employed the small-molecule 1NMPP1 obtained from Aurora Analytics, LLC (Baltimore, MD).^59^ The compound exhibited a confirmed purity of 99.2% through HPLC-UV254, and its characterization by proton nuclear magnetic resonance (400 MHz, dimethyl sulfoxide-d6 (DMSO-d6)) was consistent with its structure. A stock solution at a concentration of 200 mmol/L was prepared by dissolving 1NMPP1 in DMSO. For 1NMPP1 administration, intraperitoneal injections were conducted at 24 h and 2 h before, and 24 h after injury, utilizing a 5 mmol/L solution at a dosage of 17 μg/g body weight. DMSO-containing vehicle was employed for the control treatment. Subsequently, animals were maintained on drinking water containing 1NMPP1 (40 μmol/L in ddH2O with 1% phosphate-buffered saline (PBS)-Tween-20).

To inhibit nerve-to-HO interactions, bupivacaine liposomes (Exparel™), an FDA approved long-acting local analgesic was injected locally. Scx-eGFP mice were administered local injections of bupivacaine lipid nanoparticles (Bup-LNP, 10 mg/kg) or an equivalent volume of PBS 24 h prior to acetabular reaming. Subsequent injections were administered every 3 d for 3 weeks, guided by the drug pharmacokinetic half-life.^60^

### μCT imaging and analysis

The hip joints were harvested and fixed in 4% paraformaldehyde (PFA) at 4 °C for 24 h. Subsequently, the samples were subjected to evaluation using a high-resolution micro-CT imaging system (SkyScan 1275; Bruker, Kontich, Belgium). The scans were acquired at an image resolution of 10 μm with a 1 mm aluminum filter, X-ray voltage of 65 kVp, anode current of 153 μA, exposure time ranging from 160 to 218 ms, frame averaging set at 4, and a rotation step of 0.2°. Three-dimensional (3D) images were then reconstructed from the 2D x-ray projections using a commercial software NRecon software (v1.7.0.4, SkyScan) with the parameters: smoothing = 1, ring artifact reduction of 5%, and beam-hardening correction of 20%. The 3D reconstruction process utilized CTVox (v3.2, SkyScan). Quantification was performed using CTAn software (v1.16, SkyScan) based on volumes of interest (VOI) encompassing the newly formed bone of the femur, acetabulum, and muscular structures while excluding native bony elements. Bone volume (BV) and fractional bone volume (BV/TV), trabecular thickness (Tb.Th), trabecular number (Tb.N), and trabecular separation (Tb.Sp) were calculated from binary x-ray images using a threshold value of 70 to 255.

### Histology and immunohistochemistry

After fixation, the tissues underwent a 2 h rinse with PBS and were subjected to decalcification in 14% ethylenediaminetetraacetic acid (EDTA) (1:20 volume, Sigma-Aldrich) for 21–28 d at 4 °C. For cryosections, the tissues were cryoprotected overnight in 30% sucrose at 4 °C before being embedded in optimal cutting temperature compound (OCT) (Tissue-Tek 4583, Torrance, CA). Axial cross-sections of the hip joint, spanning from the proximal iliac bone to the distal ischium bone, were obtained using cryosections with a thickness of 30 μm. These axial sections were then mounted on adhesive slides (Fisherbrand Superfrost Plus, Thermo Fisher Scientific, Waltham, MA). Histochemical staining, including routine Hematoxylin and Eosin (H&E) and Safranin-O/Fast green (Saf-O/Fast green), or Alcian Blue and Alizarin Red Staining was performed.^13,61,62^

For immunohistochemical staining, slides were washed in PBS three times for 15 min and permeabilized with 0.3% Triton X for 30 min. Sections were subsequently blocked with SuperBlock Blocking Buffer (Thermo Scientific™, Cat# 37515) for 30 min at room temperature (RT) and then incubated with primary antibodies overnight at 4 °C. The following day, slides were washed in PBS, incubated with the appropriate secondary antibodies for 1 h at RT, and finally mounted with 4′,6-diamidino-2-phenylindole mounting solution (Vectashield H-2000, Vector Laboratories, Burlingame, CA). Images were captured using upright light and fluorescence microscopy (Leica DM6, Leica Microsystems, Inc., Buffalo Grove. IL). Digital images of these sections were further documented with confocal microscopy (Zeiss LSM900 FCS, Carl Zeiss Microscopy GmbH, Jena, Germany). Refer to Table S1 for antibody details.

### Histologic image analysis and histomorphometry

All images for quantification were obtained either with upright fluorescent microscopy (Leica DM6, Leica Microsystems Inc.) or confocal microscopy (Zeiss LSM900 FCS, Carl Zeiss Microscopy GmbH, Jena, Germany) centering around the hip HO site. Three-dimensional volumetric analysis was carried out using Imaris software v.10.0 (Oxford Instruments, Belfast, UK) for immunostaining of TUBB3, PGP9.5, TH, pSMAD2, pERK1/2 expression and co-expression of CD86 and F4/80 or CD206 and F4/80. For Cartilage and Bone Area assessments, slides were stained with Safranin O-Fast Green or Alcian Blue and Alizarin Red Staining, and analyses were conducted using ImageJ software (National Institutes of Health, MD, USA).

### Statistics

The quantitative data are presented as the mean ± 1 SD, with individual data points illustrated. Normality testing was conducted using the Shapiro–Wilk test for all datasets. Parametric data were subjected to analysis using a two-sided Student’s *t* test when comparing two groups or one-way ANOVA followed by post hoc Dunn’s multiple comparisons test when comparing more than two groups. All statistical analyses were executed using GraphPad Software 9.1 (GraphPad Software, San Diego, California). Significance levels were set at *P < 0.05, **P < 0.01, and *** *P* < 0.001.

## Supplementary information

Supplementary Materials

## Data Availability

The data that supports the findings of this study are available in the Supplementary Material of this article.
