# Supplementary material for: Pharmacologic or genetic targeting of peripheral nerves prevents peri-articular traumatic heterotopic ossification
Source: Bone Res. 2024 Sep 26;12:54. doi: 10.1038/s41413-024-00358-0 (PMC11427465; doi:10.1038/s41413-024-00358-0)
Supplement: Supplementary file 1 — Supplementary Materials [file 41413_2024_358_MOESM1_ESM.docx]

**Supplementary Materials**

**Supplementary Figure S1**


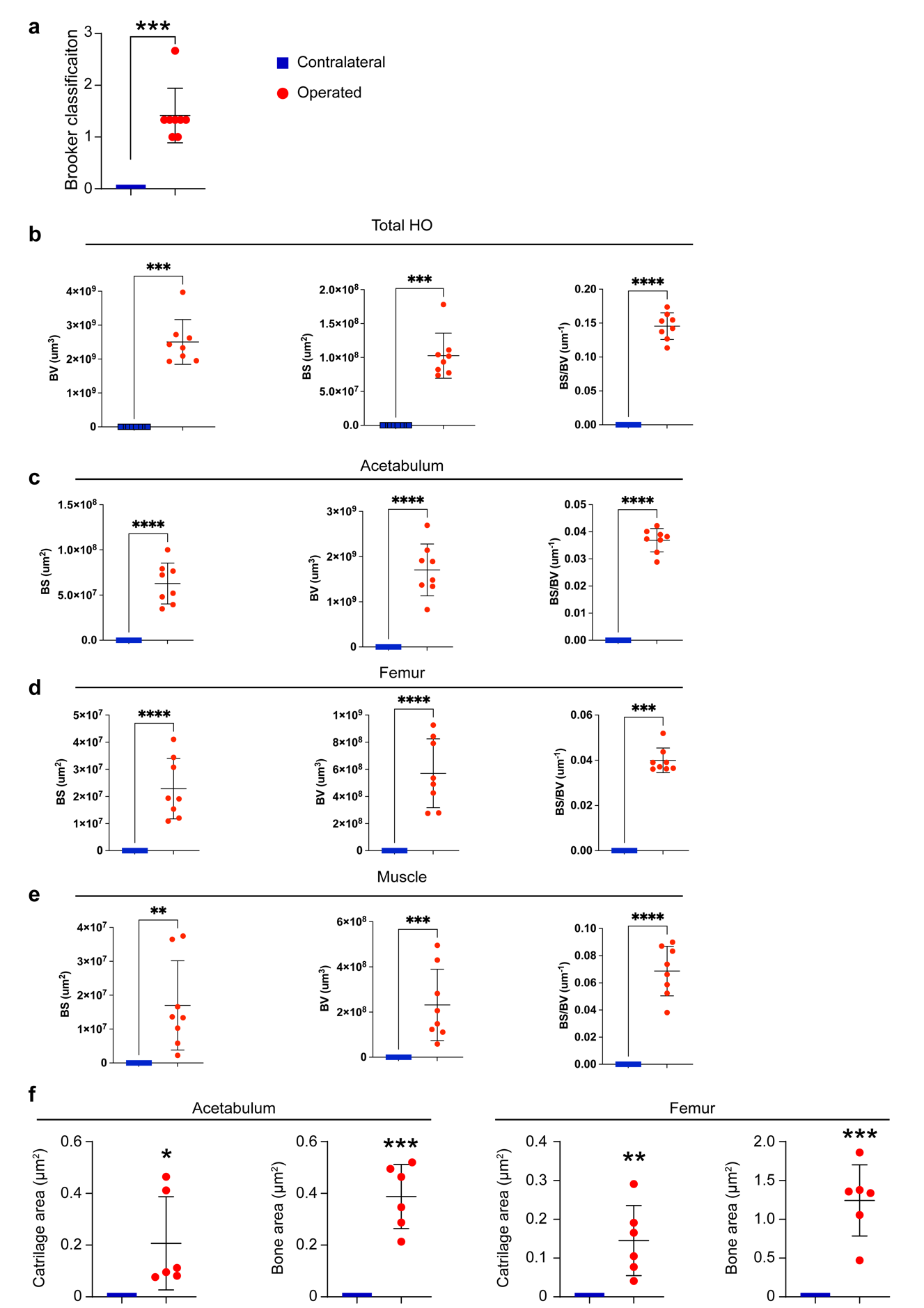


**Supplementary Figure S1.** Quantitative analysis of the hip joint at 3 wks post-surgery, Injured side is quantified in comparison to contralateral uninjured side. **(a)** Grading of HO according to a modified Brooker classification. **(b)** Quantitative μCT analysis of the hip joint, including bone surface (BS), bone volume (BV), BS/BV. **(c)** Quantitative μCT analysis of acetabulum associated HO. **(d)** Quantitative μCT analysis of femur associated HO. **(e)** Quantitative μCT analysis of intramuscular HO. **(f)** Quantification of bone and cartilaginous area within the HO site. N=8 mice per group in A-E; N=6 mice per group in F. Data presented as mean ± 1 SD. Dots in scatterplots represent an individual measurement. Unpaired two-tailed Student t-test was used for a two-group comparison. *P<0.05, **P<0.01, and ***P<0.001.

**Supplementary Figure S2**


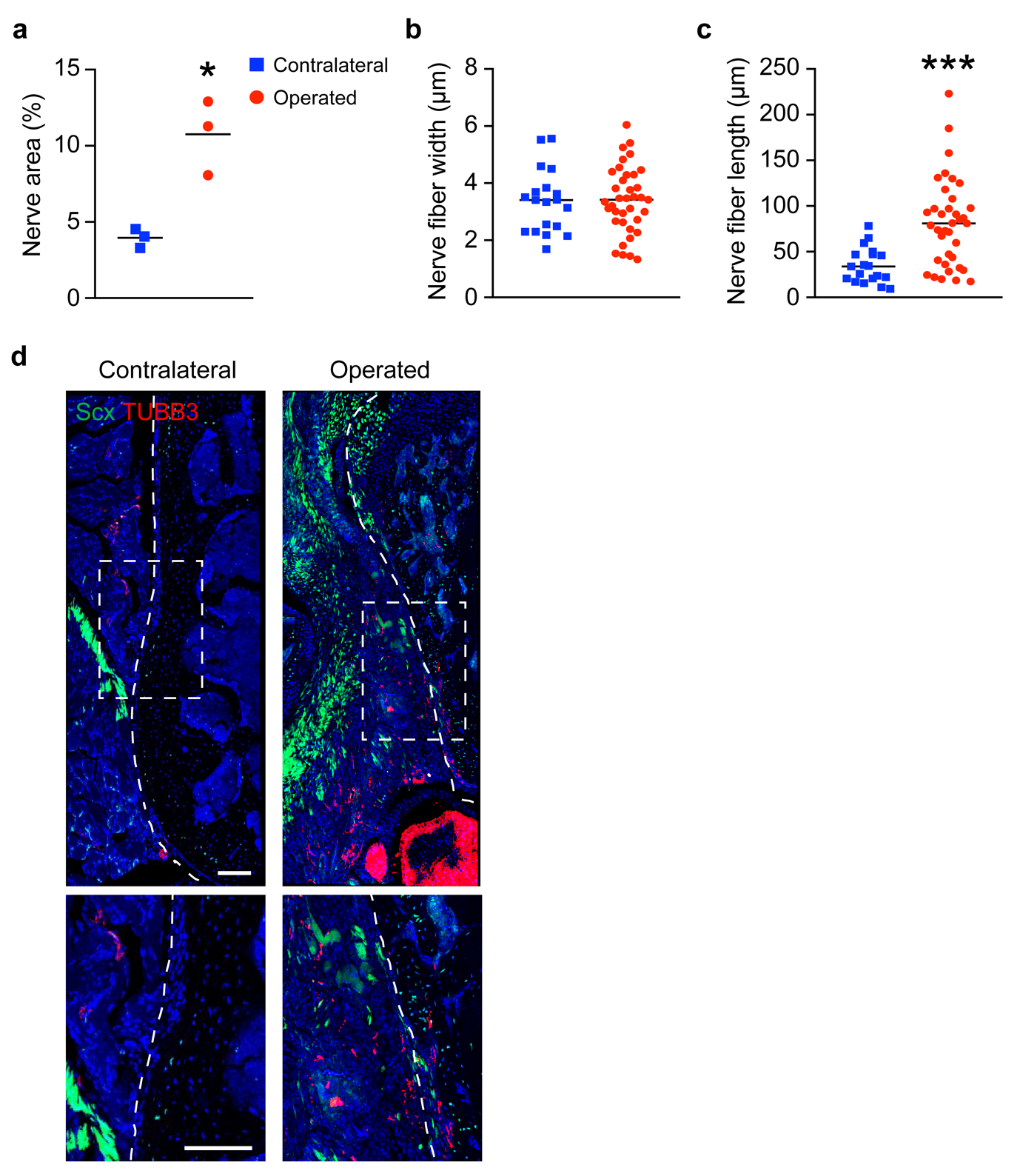


**Supplementary Figure S2.** Axonal invasion analysis after 3 weeks acetabular reaming. **(a)** Quantification of TUBB3^+^ nerve percentage area. Dots in scatterplots represent an individual joint. N = 3 animals per group. **(b, c)** Quantification of TUBB3^+^ nerve fiber width and length after 3 weeks acetabular reaming. Each dot in scatterplots represents an individual nerve detected. N = 3 animals per group. **(d)** Co-localization of TUBB3^+^ nerve and Scx^+^ cells within acetabulum HO. Data presented as mean. *P<0.05, **P<0.01, and ***P<0.001

**Supplementary Figure S3**


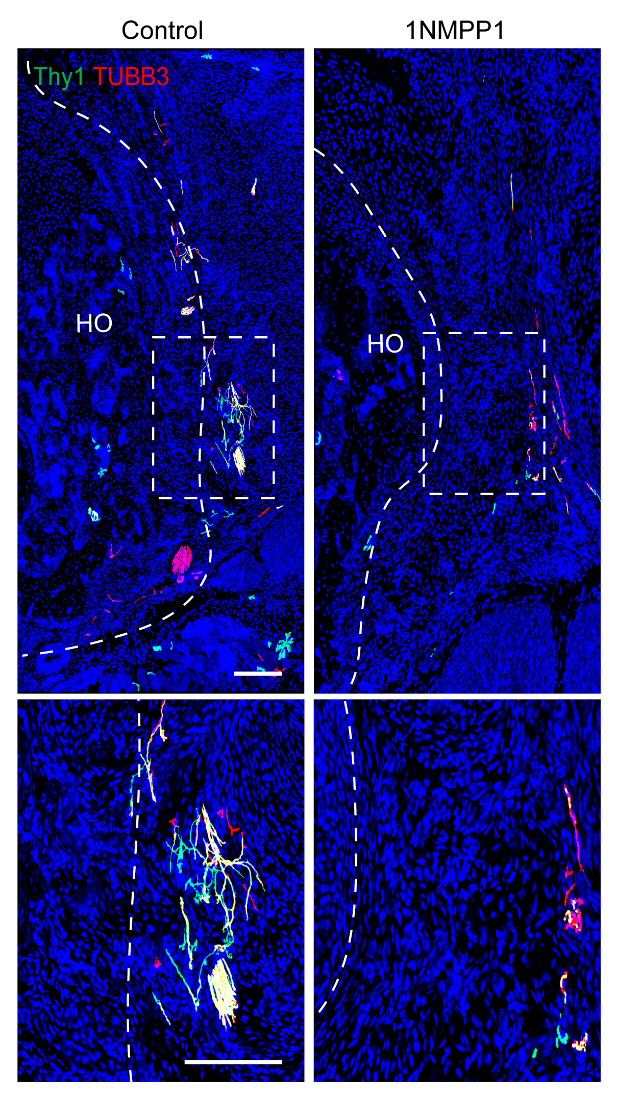


**Supplementary Fig. S3.** Representative image of TUBB3^+^ nerve staining on TrkA^F592A^/Thy1-YFP reporter mice with or without 1NMPP1 treatment. Images shown 3 wks post-operative.

**Supplementary Figure S4**

**
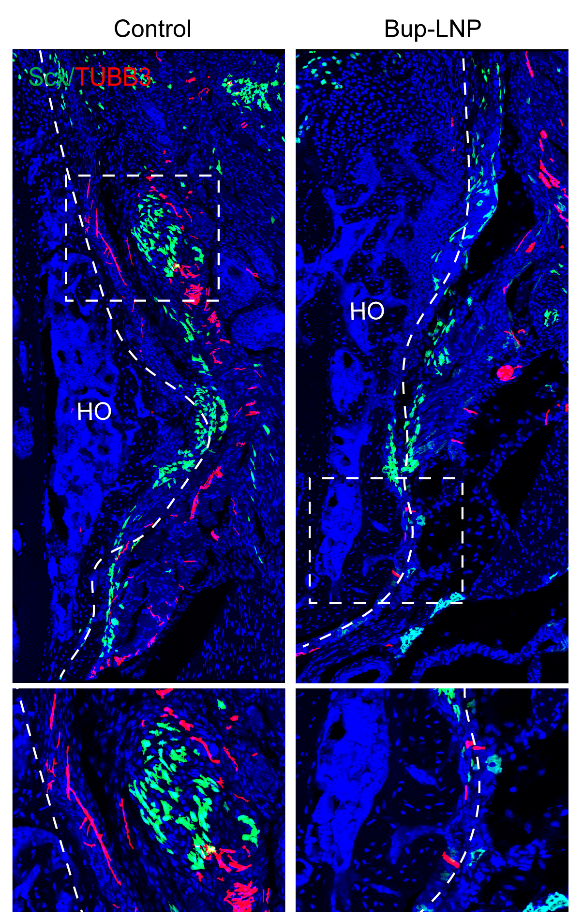
**

**Supplementary Figure S4.** Representative image of TUBB3^+^ nerve staining with Scx-GFP reporter activity in mice treated with Bup-LNP or vehicle control. Images shown 3 wks post-operative.

**Supplementary Figure S5**


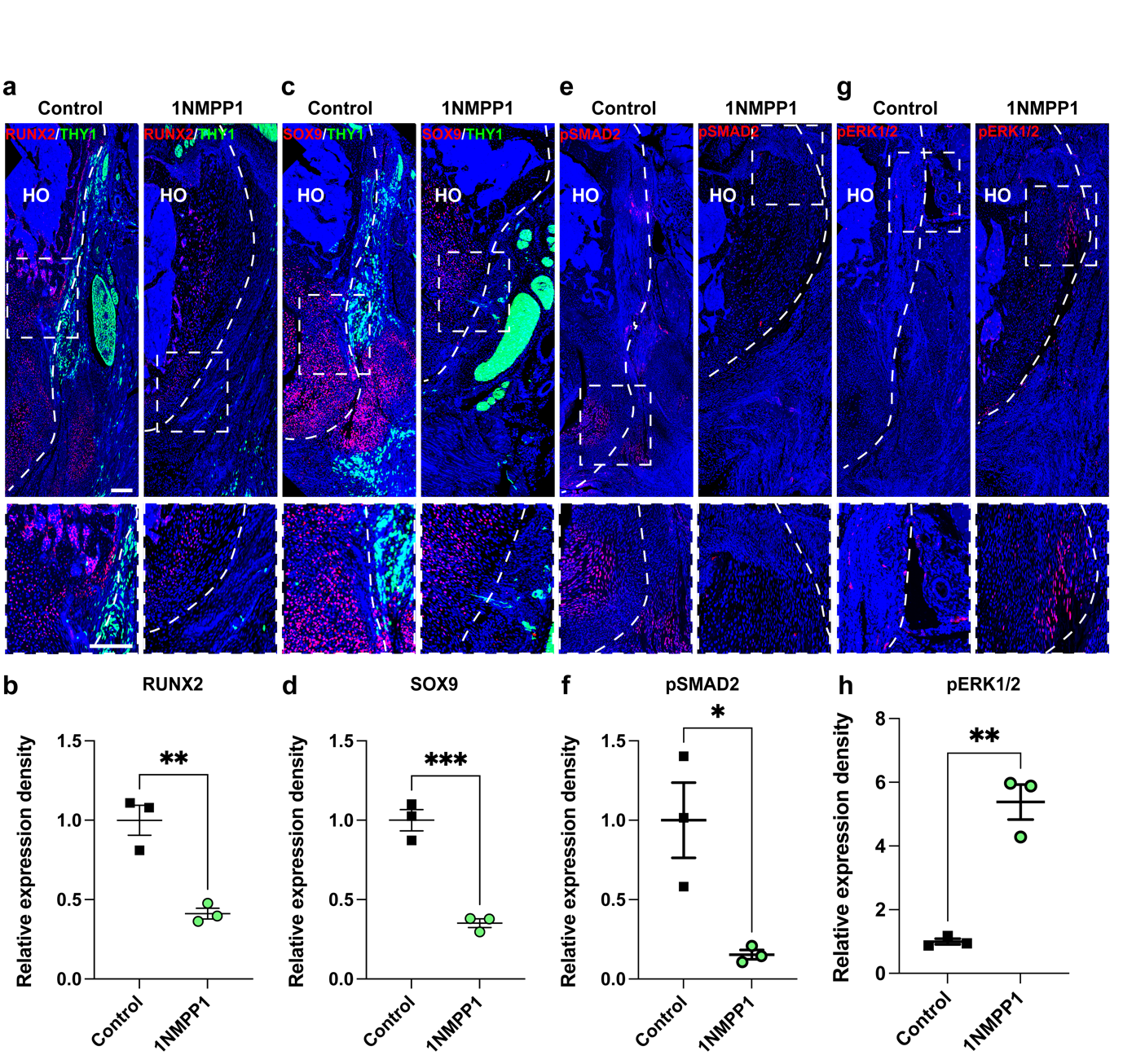


**Supplementary Figure S5.** Osteochondral changes and signaling pathways in late HO (7 weeks after acetabular remaining). TrkA^F592A^/Thy1-YFP reporter mice with or without 1NMPP1 treatment was used and examined after 7 weeks acetabular reaming. TrkA^F592A^ animals underwent HO induction, receiving either 1NMPP1 or vehicle control by i.p. injection 24 h and 2 h pre-operation and 1NMPP1 by drinking water throughout the study. **(a-d)** Representative images and quantification of the RUNX2 and SOX9 immunostaining. **(e-h)** Representative images and quantification of the pSMAD2 and pERK1/2 immunostaining. Scale bars: 200 μm. Data presented as mean ± 1 SD. Dashed lines indicate acetabulum associated HO site. Tile scans appear above, while high magnification images are below. Dots in scatterplots represent an individual measurement. Unpaired two-tailed Student t-test was used for a two-group comparison. *P<0.05, **P<0.01, ***P<0.001.

**Supplementary Figure S6**


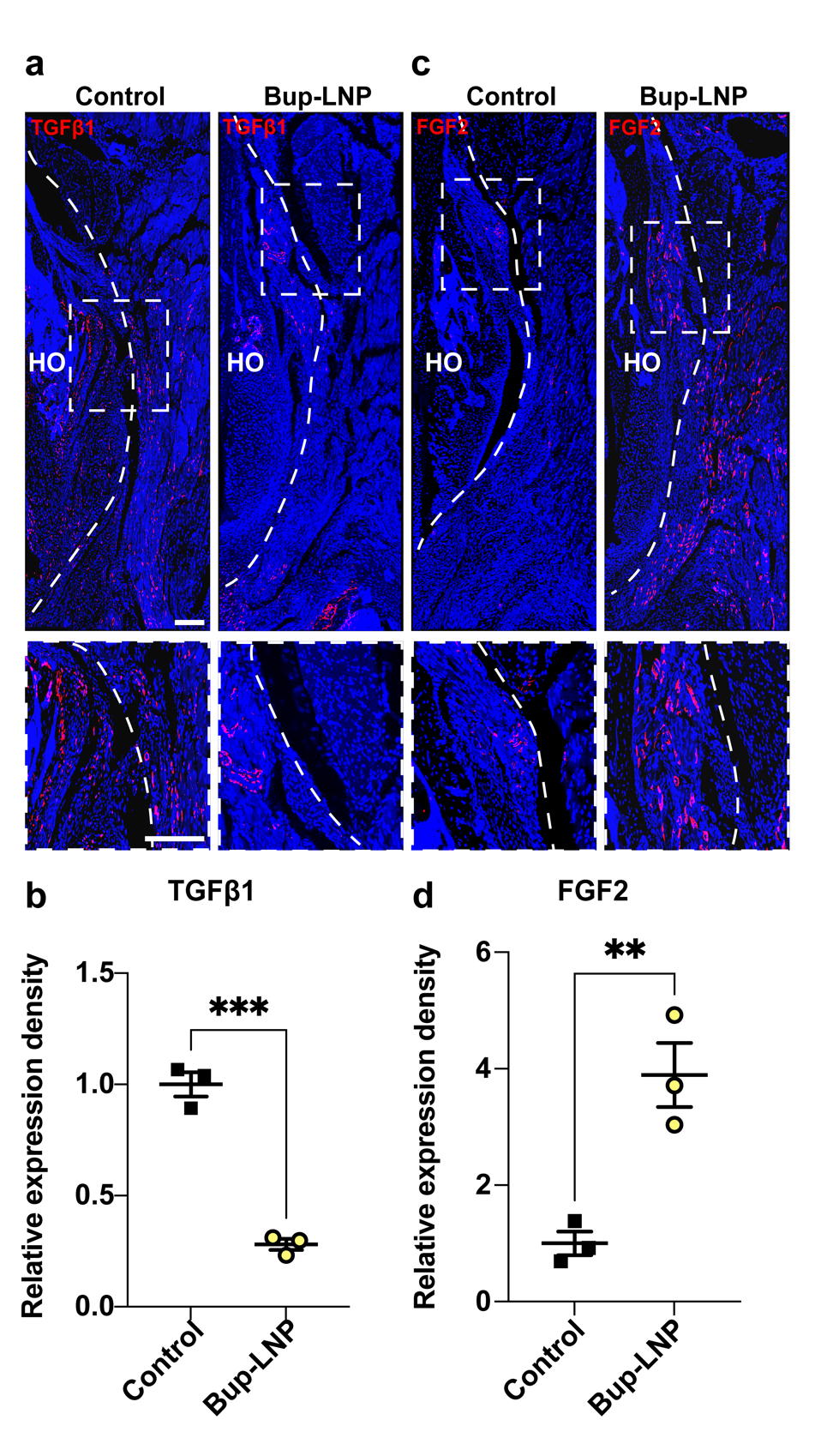


**Supplementary Figure S6.** TGFβ and FGF staining in mice treated with Bup-LNP or vehicle control. Images shown 3 wks post-operative. **(a-b)** Representative images and quantification of the TGFβ1 immunostaining. **(c-d)** Representative images and quantification of the FGF2 immunostaining. Scale bars: 200 μm. Data presented as mean ± 1 SD. Dashed lines indicate the acetabulum associated HO site. Tile scans appear above, while high magnification images are below. Dots in scatterplots represent an individual measurement. Unpaired two-tailed Student t-test was used for a two-group comparison. **P<0.01, ***P<0.001.

**Supplementary Figure S7**


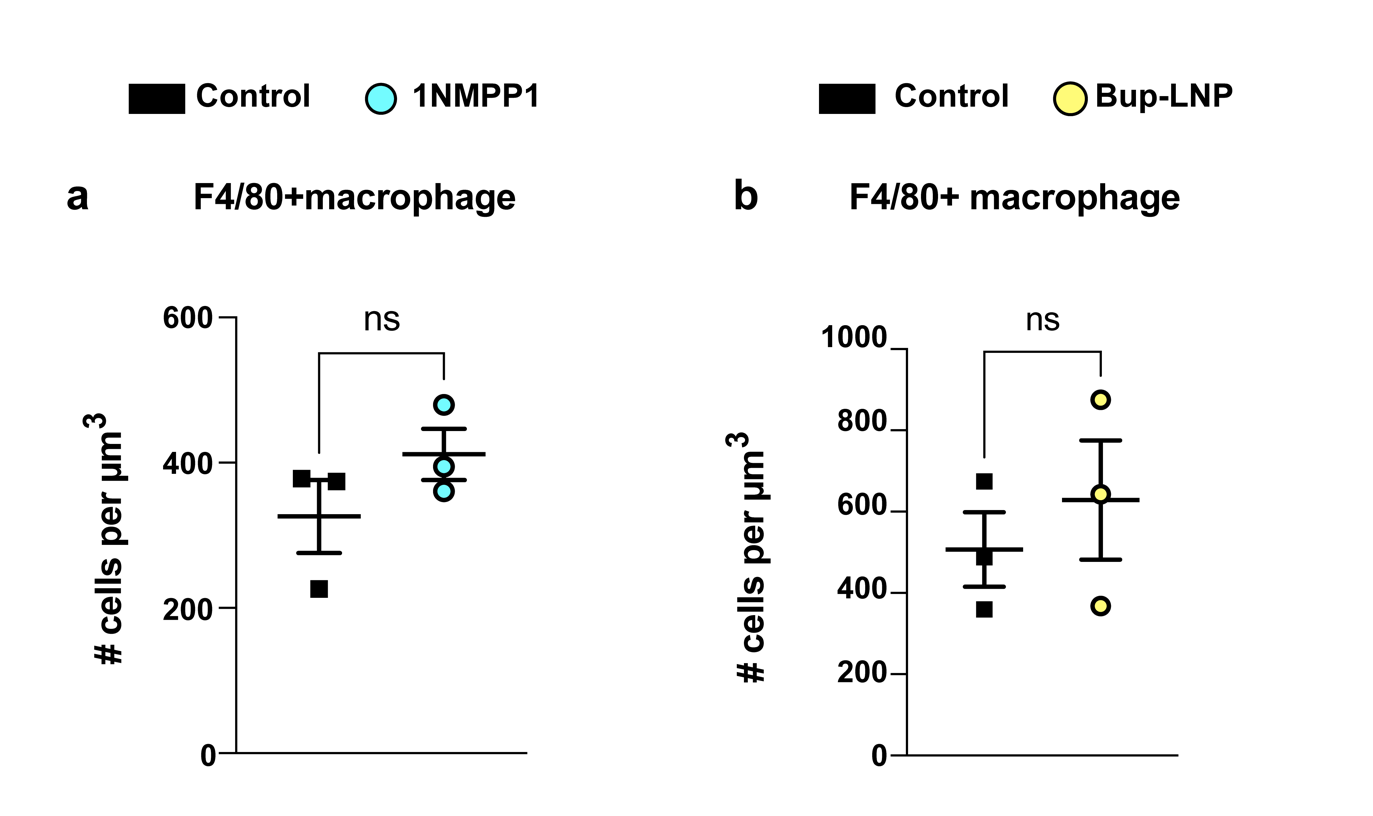


**Supplementary Fig. S7.** Quantification of F4/80^+^ macrophage density within the acetabulum-associated HO site, 3 wks post acetabular reaming. (**a**) TrkA^F592A^ mice treated with vehicle control or 1NMPP1. (**b**) Mice treated with Bup-LNP or PBS control. Dots in scatterplots represent an individual mouse measurement. N=3 animals per group. ns: non-significant.

**Supplementary Table S1.** Antibodies used.

| **Antibody** | **Company** | **Catalog #** | **Use** |
| --- | --- | --- | --- |
| Mouse Alexa Fluor 647 anti-Tubulin β3 (TUBB3) | Biolegend | 801209 | IF |
| Mouse anti-RUNX2 | Abcam | ab76956 | IF |
| Rabbit anti-TH | Sigma Aldrich | AB152 | IF |
| Rabbit anti-SOX9 | Abcam | ab185230 | IF |
| Rabbit anti-PGP9.5 | Dako | Z5116 | IF |
| Rabbit anti-NGF | Abcam | ab6199 | IF |
| Rabbit anti-pSmad2(Ser465/467) | Cell Signaling Technology | 3108 | IF |
| Rabbit anti-pERK1/2 | Cell Signaling Technology | 9101S | IF |
| Rat anti-F4/80 | Abcam | ab6640 | IF |
| Mouse anti-CD86 | Abcam | ab220188 | IF |
| Rabbit anti-CD206 | Abcam | ab64693 | IF |
| Rabbit anti-TGFβ1 | Fisher Scientific | PA1-29032 | IF |
| Rabbit anti-FGF | Fisher Scientific | PA5-116495 | IF |
| Goat Anti-Rat AF568 | Abcam | ab175476 | IF |
| Goat anti-Rabbit AF488 | Abcam | ab150077 | IF |
| Goat anti-Rabbit AF647 | Abcam | ab150115 | IF |
| Goat anti-Mouse AF594 | Abcam | ab150116 | IF |
| Goat anti-Rabbit DyLight 594 | Vector Laboratories | DI-1594 | IF |
| Goat anti-Rabbit AF647 | Abcam | ab150079 | IF |
| IF: Immunofluorescent staining. | | | |
